# Translational Research in Complementary and Alternative Medicine

**DOI:** 10.1155/2013/296817

**Published:** 2013-10-09

**Authors:** Wei Jia, Martin Kohlmeier, Aiping Lu, Rong Zeng

**Affiliations:** ^1^Cancer Epidemiology, University of Hawaii Cancer Center, Honolulu, HI 96813, USA; ^2^UNC Nutrition Research Institute, University of North Carolina Schools of Medicine and Public Health, North Carolina Research Campus, Kannapolis, NC 28081, USA; ^3^School of Chinese Medicine, Hong Kong Baptist University, Kowloon Tong, Hong Kong; ^4^Institute of Biochemistry and Cell Biology, Shanghai Institutes for Biological Sciences, Chinese Academy of Sciences, 320 Yue Yang Road, Shanghai 200031, China

Due to the compartmentalization within scientific disciplines and the fact that modern scientific methods are extensively used in CAM research, it appears that there has been a huge disconnect between clinical studies and preclinical studies including authentication, quality control, pharmacology, and toxicology of CAM agents. In this special issue, we would like to promote a concept of “translation” in CAM research by bringing a cluster of translational work (25 papers) that utilized multidisciplinary teams and approaches towards a clear clinical goal.

One of the highlights in this special issue is that there are a number of articles describing or summarizing novel approaches that may address bottleneck issues in CAM research. K. Lan et al. propose a novel strategy to determine the pharmacokinetics of multicomponent pharmaceuticals, termed as polypharmacokinetics, where the dynamic concentration profile of bioavailable xenobiotics and metabolic response profile in animals are integrated. The application of this strategy may lead to the direct elucidation of the pharmacological and molecular mechanisms of the multicomponent herbal medicines. L. Wang et al. propose an expert consensus approach that can be applied in the clinical treatment of complex diseases using traditional Chinese medicine (TCM). In their study, a group of clinical experts were consulted three times with the use of TCMs to treat hypertension, which enables investigators to take advantage of both research and clinical experience of the experts while using a standard “typical symptoms” instead of classical pattern differentiation methods. To the same goal but with differ rent approaches, J. Dai et al. introduce a macro-micro approach that combines pattern differentiation, clinical indicators, and metabolite markers to diagnose HBV-induced chronic hepatitis and nonalcoholic fatty liver disease.

More novel approaches are presented by Y. Gu et al., who propose a network flux model, using multitarget docking and network analysis, to screen molecules for antiplatelet aggregation, and X. Li et al., who provide a metabolomics-based approach to enhance the current quality control techniques for multicomponent herbal medicines.

T. Chen et al. evaluate various bioinformatics classifiers that are currently used in clinical-metabolomics studies and provide an expert opinion on the selection of classification tools based on their experimental evidence. Meanwhile, B. Zhao et al. introduce a novel strategy, in which stable-isotope labeled amino acids in cell culture were used as internal standards for clinical proteomic study, to achieve accurate quantitation of serum or urinary proteins.

Another unique feature of this issue is the extensive use of omics technologies in clinical and preclinical studies, highlighting the promise of dynamic and multiparametric profiling approach in CAM research. C. Lu et al. report a metabolomics study of hand-foot-and-mouth disease (*n* = 18), which reveals perturbation in lipid metabolism and inflammatory response in patients and showed beneficial effect of a combination therapy. Y. Zhang et al. report a metabolomics study of human aromatherapy (*n* = 31) which has, for the first time, captured the subtle metabolic changes resulting from exposure to essential oils. X. Xin et al. conduct a metabolomics study which assess the holistic efficacy of a TCM agent, compound Danshen dripping pills, for myocardial infarction in male Sprague-Dawley rats. X. Gao et al. present a urinary metabolomics study which reveals novel antipyretic mechanisms of a TCM drug, Qingkailing injection, in a rat model of yeast-induced pyrexia.

In the paper by G. Hegyi et al., the evidence and challenges of hyperthermia, overheating of a part of or the whole body, and oncothermia, which is a “spin-off” of the hyperthermia as a specialized complementary therapeutic modality, are discussed for clinical oncology. 

M. G. Porpora et al. introduce an observational cohort study on ovarian endometrioma with 92 Italian women using an alternative therapeutic agent, N-acetylcysteine, and suggest a clinically effective and feasible treatment of endometriosis based on the positive results observed.

S. Subenthiran et al. conduct a clinical CAM study on 228 patients with dengue fever with juice prepared from *Carica papaya* leaves and report that man platelet count in the treatment group (*n* = 111) was significantly higher than controls after 40 and 48 hours of admission.

Y. Gu et al. report an effective 8-week dietary intervention study of 53 healthy obese volunteers with very low carbohydrate diet. They conclude that the enhanced hepatic and whole-body lipolysis and oxidation may be associated with the clinical beneficial effects (weight loss and improved metabolic profile).

Two *in vitro* studies are included which test plant-derived extracts and compounds for bioactivities and/or therapeutic mechanisms. R. P. Samy et al. find most of the methanol extracts of 78 medicinal plants containing phenolic and polyphenolic compounds exhibit activity against the multidrug resistant Gram-negative and Gram-positive bacteria. J. G. Chung et al. report the antimetastatic activity of cantharidin, a derivative of Blister Beetles, on the adhesion, migration and invasion of human bladder cancer TSGH-8301 cells.

M. Zhang et al. observe the differential effects of two isoforms of alkylglycerols on obesity and insulin resistance in high fat diet fed mice, including significantly decreased bodyweight, serum levels of triglyceride, cholesterol, fasting glucose, insulin, and leptin by one form of alkylglycerols, and selachyl alcohol, but increased fasting insulin level by administration of the other form, batyl alcohol.

M. Wang et al. evaluate a TCM preparation, *Jiang-Zhi Granule*, on high fat diet induced steatosis in Sprague-Dawley rats and report an antisteatotic effect with a molecular mechanism through inhibiting LXRa-mediated SEBP-1c transcription and the maturation of SREBP-1c independent of LXRa.

N. Zheng et al. provide an overview of an ancient TCM, *Xiao Chai Hu Tang*, and each single herb used in the formula, for the treatment of chronic liver disease with a focus on hepatocarcinoma.

C.-J. Lin et al. report a significant preventive effect of an ancient TCM, *Bai-Hu-Tang*, in an experimental model of sepsis in male Sprague-Dawley rats, highlighting the complementary treatment option with this TCM agent for clinical sepsis.

Several studies are included in this issue evaluating traditional medicines for improving neurological conditions. E.-Y. Jung et al. report a neuroprotective effect of a traditional herbal preparation, *Gugijihwang Tang*, in a trimethyltin-induced memory dysfunction rat model. Z.-G. Yao et al. report the significant therapeutic effect of an ancient TCM preparation, PN-1, the name and the ingredients of which are not released, on the learning and memory in a transgenic mouse model of Alzheimer's disease. D. Wan et al. use a phytochemical compound, catalpol, an iridoid glycosides compound extracted from *Rehmannia glutinosa *Libosch, to treat permanent middle cerebral artery occlusion mice model and report significant neuroprotective and memory enhancement effects of this molecule. E. J. Yang and S.-Mi. Choi found that bee venom treatment attenuates the dysfunction of the ubiquitin-proteasomal system in a symptomatic hSOD1^G93A^ mice model of amyotrophic lateral sclerosis and suggest that this treatment may reduce motor neuron loss caused by misfolded protein aggregates in the mouse model.

In summary, these 25 papers represent exciting CAM research activities with translational strategies embedded in design and context. The articles cover a wide variety of topics, from novel modalities used for clinical studies to omics technologies and bioinformatics that will contribute to an improved understanding of mechanisms and pharmacology of the CAM treatments. We would like to thank all the authors and reviewers.

*Wei Jia* *Wei Jia* *Martin Kohlmeier* *Martin Kohlmeier* *Aiping Lu* *Aiping Lu* *Rong Zeng* *Rong Zeng*

